# Saturating growth rate against phosphorus concentration explained by macromolecular allocation

**DOI:** 10.1128/msystems.00611-23

**Published:** 2023-08-29

**Authors:** Gabrielle Armin, Jongsun Kim, Keisuke Inomura

**Affiliations:** 1 Graduate School of Oceanography, University of Rhode Island, Narragansett, Rhode Island, USA; 2 School of Earth, Environmental, and Marine Sciences, University of Texas Rio Grande Valley, Brownsville, Texas, USA; Ocean University of China, Qingdao, Shandong Province, China

**Keywords:** Monod kinetics, phytoplankton, macromolecular allocation, nutrient, growth, protein, carbohydrate, lipid, DNA, RNA, nutrient storage

## Abstract

The saturating relationship between phytoplankton growth rate and environmental nutrient concentration has been widely observed, yet the mechanisms behind the relationship remain elusive. Here, we use a mechanistic model of phytoplankton and show that the saturating relationship between growth rate and phosphorous concentration can be interpreted by intracellular macromolecular allocation. At low nutrient levels, the diffusive nutrient transport linearly increases with the phosphorous concentration, while the internal phosphorous requirement increases with the growth rate, leading to a non-linear increase in the growth rate with phosphorous. This increased phosphorous requirement is due to the increased allocation to biosynthetic and photosynthetic molecules. The allocation to these molecules reaches a maximum at high-phosphorous concentration, and the growth rate no longer increases despite the rise in phosphorous concentration. The produced growth rate and phosphorous relationships are consistent with the data of phytoplankton across taxa. Our study suggests that the key control of phytoplankton growth is internal, and nutrient uptake is only a single step in the overall process.

**IMPORTANCE:**

The Monod equation has been used to represent the relationship between growth rate and the environmental nutrient concentration under the limitation of this respective nutrient. This model often serves as a means to connect microorganisms to their environment, specifically in ecosystem and global models. Here, we use a simple model of a marine microorganism cell to illustrate the model’s ability to capture the same relationship as Monod, while highlighting the additional physiological details our model provides. In this study, we focus on the relationship between growth rate and phosphorus concentration and find that RNA allocation largely contributes to the commonly observed trend. This work emphasizes the potential role our model could play in connecting microorganisms to the surrounding environment while using realistic physiological representations.

## INTRODUCTION

Phytoplankton are responsible for most primary production and photosynthesis in the ocean (1, 2). They are also at the core of global biogeochemical cycles and the oceanic, biological carbon pump (3, 4), the magnitude of which is strongly influenced by the phytoplankton growth rate (5, 6). Nutrient fluxes of nitrogen (N) and phosphorus (P) are often the major limiting factor in phytoplankton growth (7 – 9). Due to the altered nutrient supply, environmental changes, including eutrophication and climate change, have had a significant effect on the growth rate and size of phytoplankton (7, 10 – 12). Understanding the growth rate of phytoplankton and their response to respective nutrient concentrations provides insights into physiological responses at the cellular level (13). Therefore, the relationship between phytoplankton growth rate and nutrient concentration in the ocean is critical to understand their role in marine ecosystems. One such model that quantifies this relationship is the Monod kinetic model which describes the growth rate of phytoplankton (*μ*) as a function of nutrient concentration following (Eq. 1) (14).


(Eq. 1) $$\mu =\;\mu_{max}\frac{S}{K_{s}+S}$$

Here, $\mu_{max}$ is the maximum specific growth rate (day^−1^) of microorganisms at substrate saturation, *S* is the substrate concentration (µM), and *K_S_* is the half-saturation constant (µM) as a value of substrate concentration corresponding to half of *μ_max_*. The equation suggests that extracellular nutrient concentration is the limiting factor of phytoplankton growth rates. Subsequent studies illustrated these nutrient dependencies on growth rates by applying Monod’s theory to various marine plankton and substrates (15 – 17). Select studies demonstrated that the growth rate was affected by different phosphorous concentrations and uptake rates when testing the Monod kinetic model with various phytoplankton (18 – 20).

Still, the Monod equation is widely applied to predict the relationship of growth rate and nutrient concentrations, specifically phosphorous. Although the Monod model derives the maximum growth rate, it conveys limited physiological information. For example, it is still unclear what controls the maximum growth rate and the reason behind the saturating relationship between nutrient concentrations and the growth rate. Previous studies (21, 22) pointed to internal effects within phytoplankton as the cause of growth rate limitation, but a mechanistic model of cellular processes would offer further physiological-based predictions (23).

**Fig 1:**
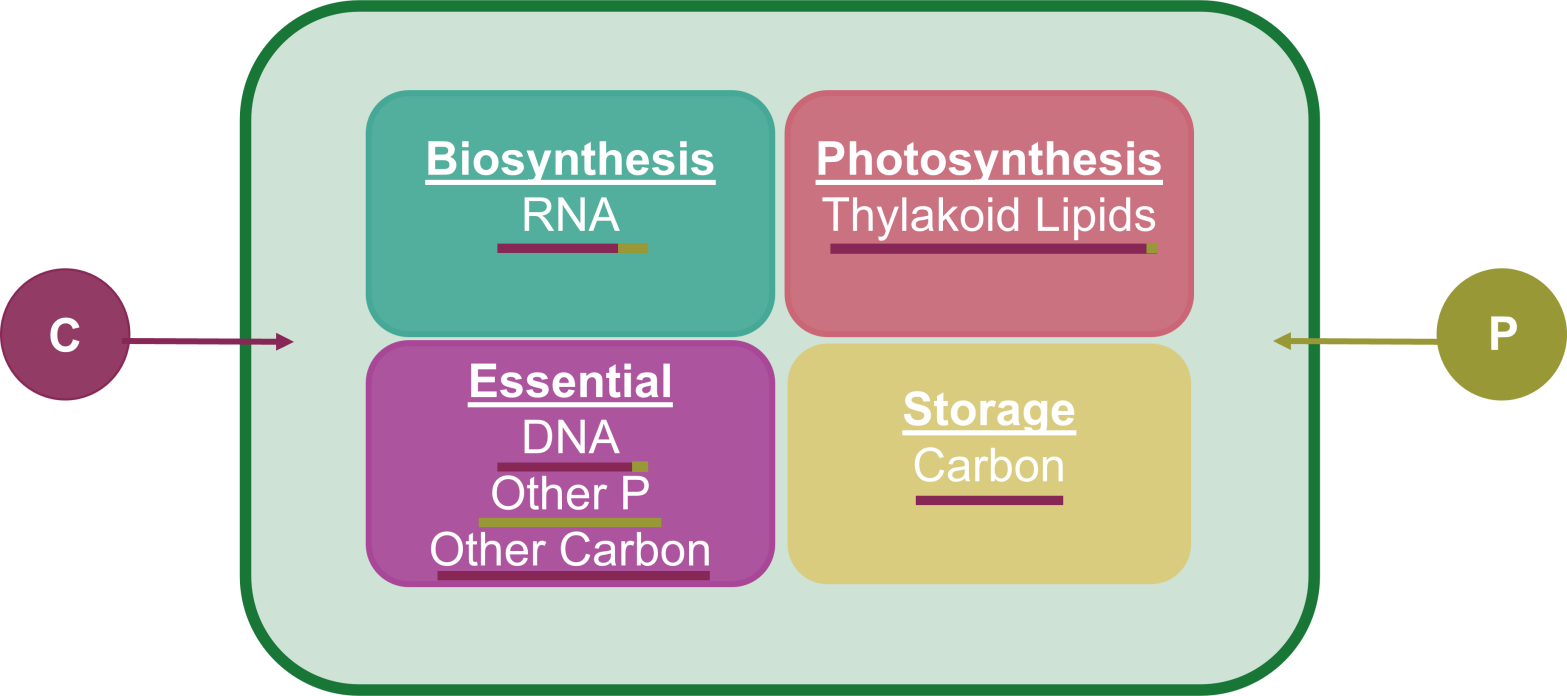
The Cell Flux Model of Phytoplankton (CFM-Phyto) under P limitation. The model allocates carbon (maroon) and phosphorus (olive) to four intracellular macromolecular pools: biosynthesis (teal), photosynthesis (pink), essential (purple), and storage (yellow). Each macromolecule requires varying levels of C and P (24), indicated by the bar below the macromolecule. When C is limited, there is no allocation of C to storage. The essential pool represents macromolecules needed for basic cell survival and structure and is assumed to remain constant with growth rate.

The Cell Flux Model of Phytoplankton (i.e., CFM-Phyto) was recently developed to explore the relationship between growth rate, elemental stoichiometry, and macromolecular allocation (e.g., proteins, DNAs, RNAs, carbohydrate, and chlorophyll) in phytoplankton (24) (Fig. 1). Subsequent studies demonstrated that CFM-Phyto provides key insights of cellular physiology in the various environments. For example, CFM-Phyto was applied to predict C:P ratios in the ocean with the satellite remote sensing data (25) and explore the temperature dependency of nutrient ratios in phytoplankton (26). A recent study also used the CFM-Phyto to understand the saturating relationship between phytoplankton growth rate and nitrogen concentration and provided macromolecular-based interpretations (27). However, there is a lack of understanding of the relationship between growth rate and phosphorous concentrations in phytoplankton from a cellular perspective, although phosphorous is one of the key elements in aquatic ecosystems (28, 29).

In this study, we focus on CFM-Phyto’s ability to provide physiological context and use it to interpret the saturating relationship between the growth rate and phosphorus concentration. We developed the model to address the following questions: (1) Can CFM-Phyto capture the saturating relationship between PO_4_^3−^ concentration and growth rate often modeled by Monod kinetics? (2) What leads to the saturating relationship between the growth rate and phosphorus concentration? (3) How does this relationship differ from that between growth rate and nitrogen concentration? Here, we focus on the relationship between the growth rate and the concentration of one of the major nutrients, PO_4_^3−^, using data of phytoplankton across taxa. The model provides a macromolecular-based interpretation of the widely observed saturating relationship.

## RESULTS AND DISCUSSION

**Fig 2:**
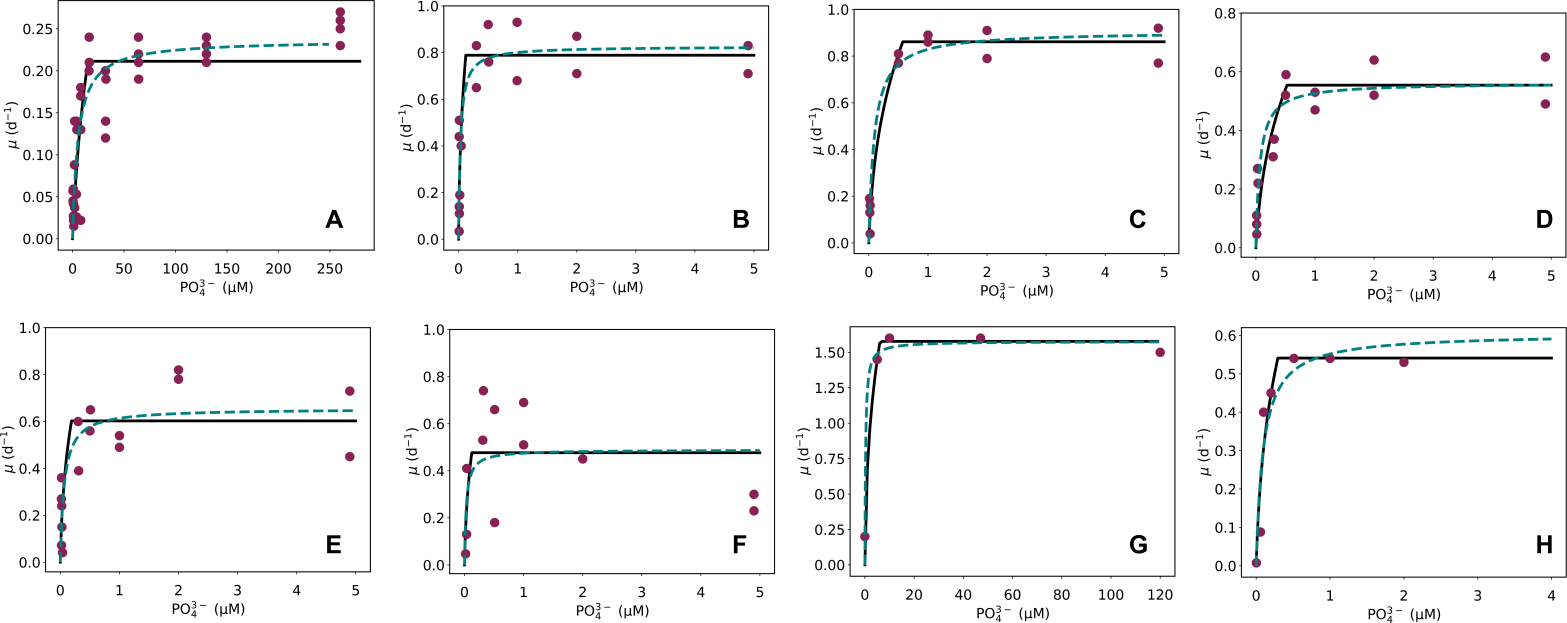
Measured (maroon points) and predicted growth rates with increasing PO_4_^3−^ using the Cell Flux Model of Phytoplankton (CFM-Phyto; black line) and the Monod formulation (dotted teal line) for various organisms (**A**) *Microcystis* (18), (**B**) *Chorella* sp. (20), (**C**) *Nitzschia palea* (20), (**D**) *Oocystis pusilla* (20), (**E**) *Scenedesmus quadricauda* (20), (**F**) *Sphaerocystis schroeteri* (20), (**G**) *Synechocystis* sp. PCC6803 (19), and (**H**) *Pelagomonas capsulatus* (17).

We compared both the Monod mathematical model and the CFM-Phyto output to experimental data of the measured growth rates with increasing PO_4_^3−^ concentrations for 15 organisms (Fig. 2; Fig. S1). Here, we define two major phases from the model output: a rapidly increasing growth rate followed by a constant growth rate. During the sharp increase, P availability limits growth, while C availability limits growth during the second phase. Similar to an N-limited environment (27), the addition of C limitation to the model forces the growth rate to reach a saturation point rather than continuously increasing as illustrated in the Monod mathematical model, as shown in most data (Fig. 2; Fig. S2). By imposing a maximum growth rate, and in turn, a maximum amount of cellular phosphorus, CFM-Phyto realistically represents nutrient dynamics within a cell. One mechanism that neither Monod nor CFM-Phyto captures is the growth inhibition at high concentrations of phosphorus observed in *Sphaerocystis schroeteri* (Fig. 2F) and *Synedra radians* (Fig. S1E). The mechanism behind the growth inhibition is unknown. A possible mechanism may include negative feedback. Also, the data are not based on the axenic culture, and the high phosphorus concentration might have induced the growth of other organisms harmful to the measured organisms. Further studies need to be done to clarify the common mechanisms of such growth inhibition, which may eventually be incorporated into the modeling. In this study, the macromolecular allocation largely explains the saturating growth curve, as observed in most phytoplankton taxa.

**Fig 3:**
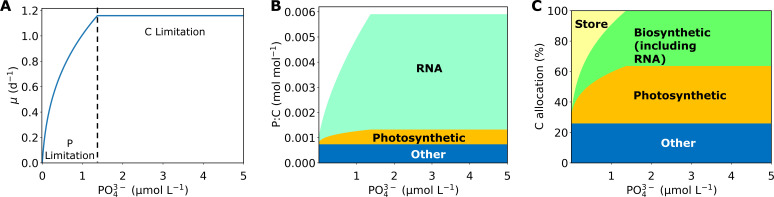
An example of simulated growth rate and macromolecular allocation produced by the cell flux model of phytoplankton (CFM-Phyto). (**A**) Growth rate. (**B**) Macromolecular allocation based on P (per cellular C). (**C**) C-based macromolecular allocation. The overall patterns of the relationships are conserved across the simulations.

Under P limitation, the cell prioritizes P use in biosynthesis, photosynthesis, and building essential biomolecules (Fig. 3) without accumulating any P in storage. Therefore, macromolecular allocation to RNA largely contributes to the observed increasing growth rate at low P concentrations. RNA ultimately controls protein synthesis which is necessary to create proteins that enhance key cellular reactions within photosynthesis and biosynthesis that produce energy for the cell, allowing it to grow. The initial investment in P uptake allows the cell to make these key biomolecules which contribute to high growth rates. As the growth rates increase, C storage depletes and eventually C limitation occurs which causes the increasing growth rate to slow to a constant value of 1.16 d^−1^. When P is the most limiting factor, the cell uses available C in storage to create photosynthetic machinery and RNA, both integral to the central metabolism of the phytoplankton cell. Once the C stores are exhausted, the cell may not grow faster with additional PO_4_^3−^ availability.

**Fig 4:**
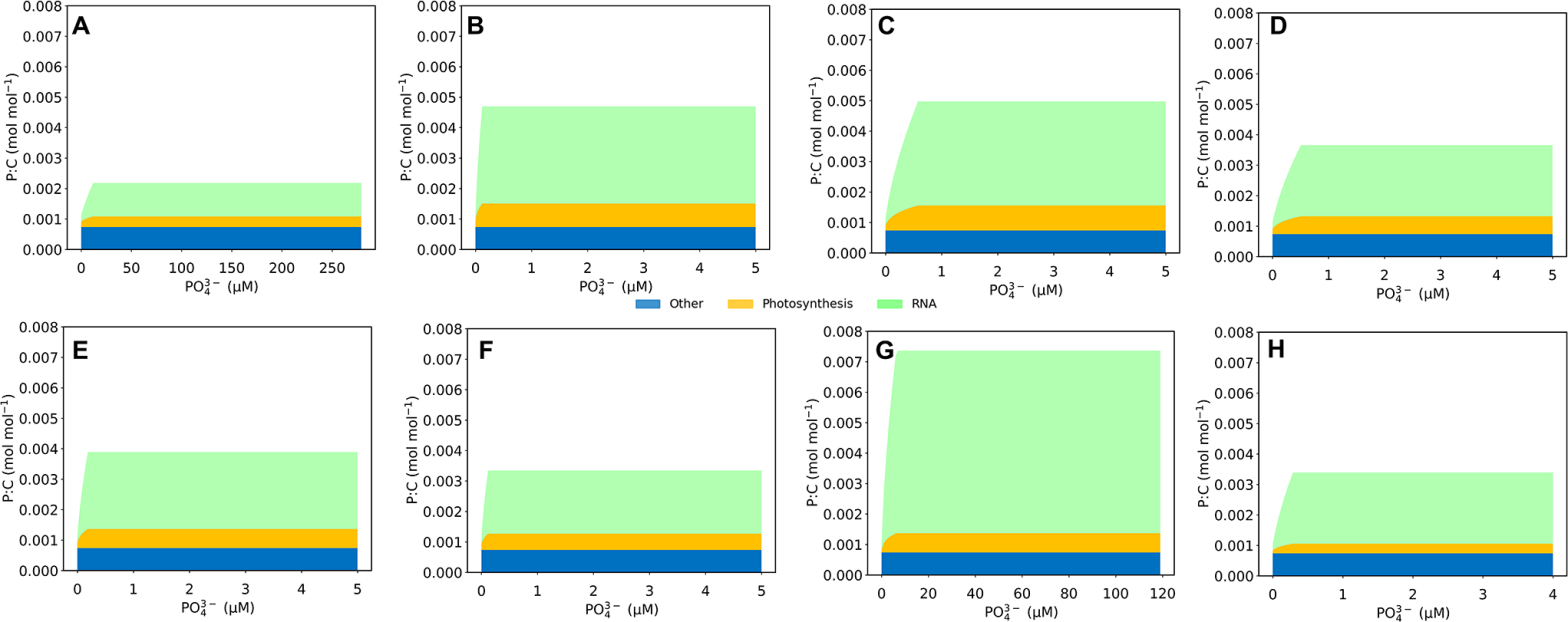
Species-specific predictions of macromolecular allocation of P to three cellular pools: RNA (green), photosynthetic molecules (orange), and other (blue). Other molecules include DNA and remaining P. (**A**) *Microcystis* (18), (**B**) *Chorella* sp. (20), (**C**) *Nitzschia palea* (20), (**D**) *Oocystis pusilla* (20), (**E**) *Scenedesmus quadricauda* (20), (**F**) *Sphaerocystis schroeteri* (20), (**G**) *Synechocystis* sp. PCC6803 (19), and (**H**) *Pelagomonas capsulatus* (17).

Generally, phytoplankton follows these patterns of P allocation, but there are some variations species-to-species (Fig. 4; Fig. S2). Specifically, the higher maximum growth rates may require more P to build RNA and photosynthetic molecules and contribute to the variation in P dedication to these macromolecules among the different phytoplankton species. Here, *Synechocystis* sp. PCC6803 (19) had the highest maximum growth rate, and accordingly, the model predicts the highest P:C value and fraction of RNA (Fig. 4G). Whereas we did not include P storage in this particular study, it is likely that such luxury uptake of P happens in many cases, when C becomes limited and thus P may no longer be limited. Data compilations so far suggest that eukaryotic cells tend to store more P for specific biomass (30 – 32). At the species level, however, the magnitude of the P storage varies, and additional measurements would be needed to constrain the P storing capacity of the individual species.

**Fig 5:**
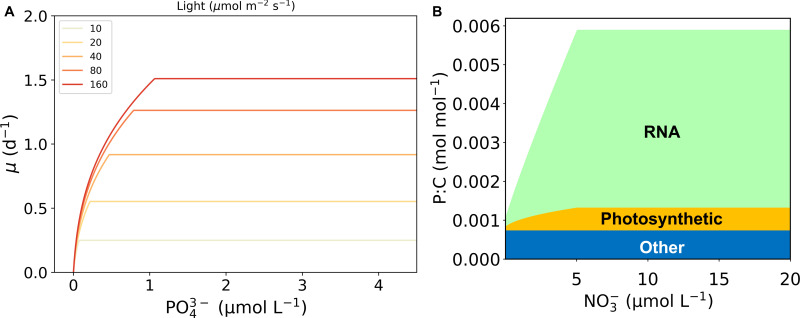
The Cell Flux Model of Phytoplankton (CFM-Phyto) predicts growth rate (*μ*) with increasing phosphate concentration (PO_4_^3−^) for various environmental conditions including light intensity. (**A**) Darker colors represent higher light intensity or temperature. CFM-Phyto also predicts macromolecular allocation of phosphorus under nitrogen limitation (**B**).

The framework of the CFM-Phyto allows for a strong connection between the phytoplankton cell and the environment by predicting maximum growth rates and macromolecular allocation of P in various environmental conditions, such as nitrogen limitation or changing light availability (Fig. 5). In reality, there are many factors that govern phytoplankton growth in the surrounding environment, and by limiting the external factors to a single stressor, the Monod model excludes other key influences. Using the CFM-Phyto illustrates that light intensity has impacts on maximum cellular growth in addition to nutrient limitations. With increasing light intensity, the maximum growth rate increases and requires a higher concentration of P to reach this maximum (Fig. 5A). Increasing light intensity, before the point of photoinhibition, allows for efficient photosynthetic activity (33 – 35), supporting fast growth in the cell, but increases the P requirement to build macromolecules such as RNA to maintain these higher growth rates. In addition to these external factors, the CFM-Phyto simulates macromolecular allocation of P under P and N limitation (Fig. 4; Fig. 5B). Higher N availability (here NO_3_^−1^) leads to higher photosynthetic and biosynthetic molecules (e.g., RNA), leading to higher allocation of P to these molecules, until the N switches to C limitation where N availability may no longer have an impact. Under both N and C limited cases, luxury uptake of P may occur, and the magnitude of the P storage would depend on multiple factors, including the availability of P, growth rate, and the individual capacity of P storage (32).

**Fig 6:**
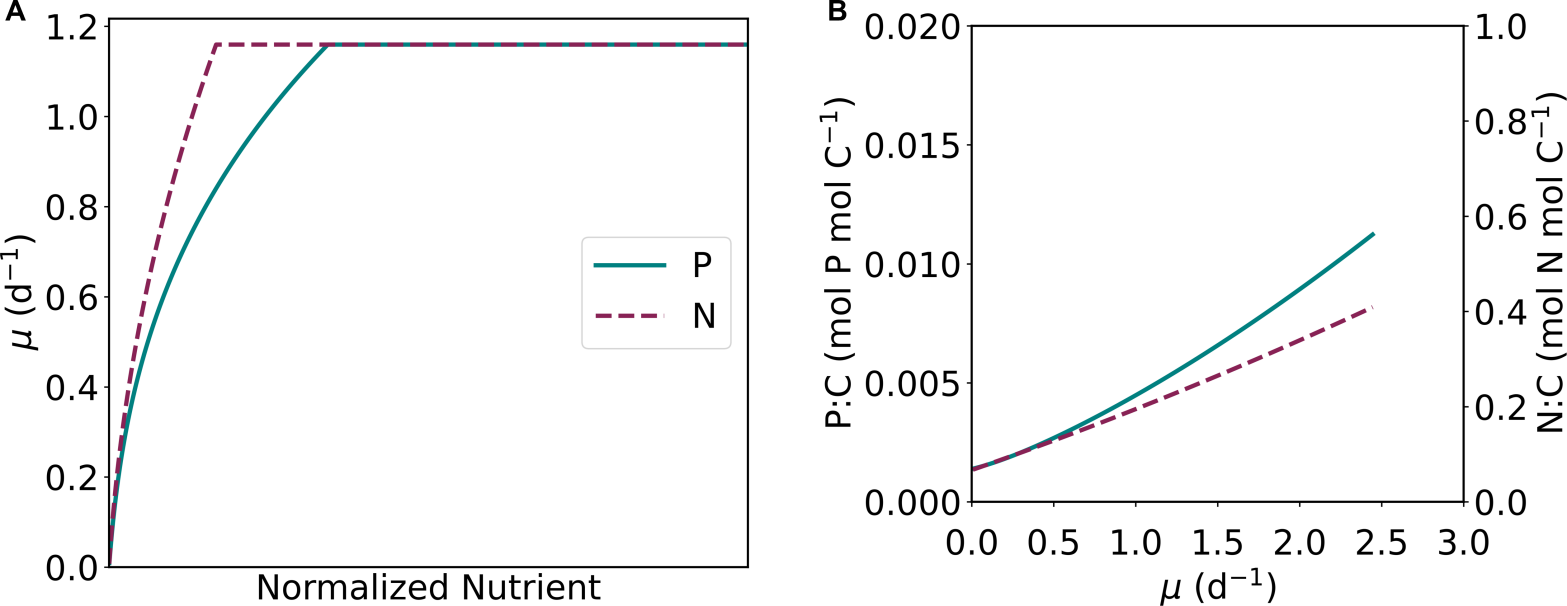
Comparison of growth with increasing nutrient concentrations (**A**) of PO_4_^3−^ (solid, teal line) and NO_3_^−^ (dashed, maroon line) for the respective nutrient limitation (i.e., P and N limitation) normalized to the cellular ratios of phosphorus using a typical nutrient ratio (30) (i.e., dividing N by 15). (**B**) Comparisons of intracellular P:C (teal) (Mol P mol C^−1^) and N:C (maroon) (Mol N mol C^−1^) for increasing growth rate.

Macromolecular allocation explains the cellular dynamics of nutrient uptake and highlights the differences between N and P. Previously, we captured a similar saturating growth curve using CFM-Phyto for increasing N concentrations (27). In both nutrient limitations (N and P), there is an increasing phase of growth, followed by a constant growth rate. Similarly, the first part is characterized by P or N limitation, where the growth rate increases with the addition of each nutrient, and the stable part is due to C limitation. The subtle, yet noteworthy, difference between the two nutrients is the rate and nature of the increase during the N or P limitation. To compare the two nutrients (Fig. 6A), we normalized the N value to P by dividing N by 15, a commonly observed N:P value in oceanic organic matter and water (30). For each nutrient, we have a unique set of equations that highlight these differences and offer a physiological explanation for the discrepancies. On the macromolecular level, P allocation is dominated by RNA, whereas N is predominantly allocated to proteins. Proteins are mainly used as enzymes that catalyze biosynthetic reactions, whereas RNA is mainly involved in protein synthesis. This difference in biochemical roles leads to the difference in the relationship between these molecules and growth rate, leading to a linear relationship between growth rate and protein and a quadratic relationship between growth rate and RNA, respectively (24, 36 – 38). Because the growth rate and the cellular content of limiting nutrient are inversely related, a quadratic relationship between the growth rate and P rich RNA leads to a more gradual change in growth rate toward C limitation relative to the initial slope under P limitation (Fig. 3B). This trend may contribute to more data points where growth rate increases with nutrient concentration under P limitation than N limitation (compare this study and reference 27).

### Conclusion

Previously, the widely used Monod mathematical formulation modeled the saturating growth rate with increasing phosphorus concentration. Here, we illustrate the strength of using CFM-Phyto to capture this trend due to its predictions of macromolecular allocation, specifically for phosphorus and carbon allocation within the cell. Physiologically, the saturating relationship between P and growth rate can be attributed to a dedication to RNA molecules and a depletion of C storage, and thus, C allocation to growth-related and essential molecules. Moreover, the allocation to RNA, rather than to proteins, explains the subtle difference between P- and N-based growth. Due to the ease the Monod model provides of relating nutrient uptake and growth, it is often used in ecosystem models to connect nutrient consumption to lower trophic organisms. However, CFM-Phyto captures the trend as well as Monod and provides additional information about cellular physiology like macromolecular allocation and may be altered to simulate other environmental conditions such as light intensity. CFM-Phyto remains a simplistic representation of phytoplankton and thus can be used in larger ecosystem models while providing more cellular predictions based on the ever-changing, surrounding environment.

## MATERIALS AND METHODS

The following details outline our use of both the Monod mathematical kinetics model and the CFM-Phyto to capture the trend between phosphorus concentration and growth rate. Not only this, but we also provide equations and assumptions that led to the physiological interpretation of this commonly observed trend. We used published data that spanned phytoplankton taxa to illustrate the versatility of the CFM-Phyto. Please see the supplemental material for a list of the data sets we used and notes about the experiments (Table S1).

### Monod kinetics

To optimize the Monod kinetics mathematical model (Eq. 1), we used the Metropolis-Hastings algorithm (39 – 41), a Markov Chain Monte Carlo (MCMC) method that introduces perturbations to our initial estimates and eventually converges to parameter values that best fit the data. We gave initial estimates for the maximum growth rate ( $\mu_{max}$ ) and the half-saturation constant (*K*_*P*_), converging on the best solution with visual trial-and-error. We recorded the resultant values of the maximum growth rate and the half-saturation constant for each data set (Table S2).

### CFM Representation

The CFM-Phyto is a coarse-grained model that predicts nutrient allocation to four categories of macromolecules including molecules involved in photosynthesis, biosynthesis, essential cellular structure and survival, and storage of nutrients (Fig. 1). Additionally, it calculates the resulting elemental stoichiometry (C:N:P). A variety of environmental conditions can be simulated using this model. CFM-Phyto is based on key assumptions that include linear relationships between the RNA, protein, and growth rate (36, 37, 42, 43), a constant macromolecular composition of the photosynthetic machinery (44 – 47), and a saturating function between irradiance and photosynthesis (48, 49). These assumptions, in addition to the following macromolecular allocation equations, comprise the model.

Again, we grouped biomolecules into four categories: photosynthesis, biosynthesis, essential, and storage (Fig. 1). Phosphorus is primarily dedicated to RNA in biosynthesis, the thylakoid membrane lipids in photosynthesis, DNA, and structural lipids in essential biomolecules, and storage. Storage of either phosphorus or carbon only occurs when that nutrient is in excess. Here, we ran the model in phosphorus limitation and carbon limitation. Therefore, carbon is only stored during phosphorus limitation, and phosphorus is only stored during carbon limitation.

For an extensive list of all equations, parameters, their respective definitions, and derivations please refer to Tables S3 and S4 in the supplemental material. Here, we highlight some key equations that informed the model output. First, we describe the equations that quantify macromolecular allocation for carbon (Eq. 2), phosphorus uptake (3 [Disp-formula uFD4] - 5), and phosphorus allocation (Eq. 6). We simplified these equations, along with those listed in the supplemental material, into two equations (7–8) and solved them for the growth rate:


(Eq. 2) $$1=Q_{C}^{Pro}+Q_{C}^{RNA}+{Q_{C}^{DNA}+Q}_{C}^{Chl}+Q_{C}^{Plip-Thy}+Q_{C}^{Csto}+Q_{C}^{Other}$$

The categories we used include proteins ( $Q_{C}^{Pro}$ ), RNA molecules ( $Q_{C}^{RNA}$ ), DNA molecules ( $Q_{C}^{DNA}$ ), chlorophyll ( $Q_{C}^{Chl}$ ), phospholipids in the thylakoid membranes ( $Q_{C}^{Plip-Thy}$ ), phosphorus carbon storage ( $Q_{C}^{Csto}$ ), and all remaining carbon labeled as other ( $Q_{C}^{Oth}$ ). We describe the change of cellular phosphorus concentration over time ( $\frac{dQ_{P}}{dt}$ ) by subtracting the phosphorus dedicated to new cell growth ( $\mu Q_{P}$ ) from the rate of nitrogen uptake ( $V_{P}$ ).


(Eq. 3) $$\frac{dQ_{P}}{dt}=V_{P}-\mu Q_{P}$$

We assumed there is no change in the cellular phosphorus concentration over time ( $\frac{dQ_{P}}{dt}=0$ ), or steady-state conditions, and (Eq. 3) becomes


(Eq. 4) $$V_{P}=\mu Q_{P}$$

where the cellular phosphorus is defined by the macromolecular allocation of phosphorus (Eq. 6). This equation assumes the rate of diffusion limits the cellular concentration of phosphate; thus the uptake is linearly related to the phosphate concentration $\left[{PO}_{4}^{3-}\right]$ with an affinity constant *A_P_* (50):


(Eq. 5) $$V_{P}=A_{P}[{PO}_{4}^{3-}]$$

We calculated phosphorus allocation using a stoichiometric ratio (24, 51) between phosphorus and carbon to convert the above carbon calculations to a value representative of phosphorus (Eq. 6).


(Eq. 6) $$Q_{P}=Q_{P}^{Thy}+Q_{P}^{RNA}+{Q_{P}^{DNA}+Q}_{P}^{Oth}+Q_{P}^{Sto}$$

The model allocates phosphorus to lipids in the thylakoid membranes ( $Q_{P}^{Thy}$ ), RNA molecules ( $Q_{P}^{RNA}$ ), DNA molecules ( $Q_{P}^{DNA}$ ), and the remaining phosphorus ( $Q_{P}^{Oth}$ ). To obtain the growth rate, we solve the relationship for both C limitation under the steady state, (Eq. 7) and (Eq. 8), respectively:


(Eq. 7) $$0=a_{C}\mu^{2}+b_{C}\mu +c_{C}$$

(Eq. 8) $$0=a_{P}\mu^{3}+b_{P}\mu^{2}+c_{P}\mu +d_{P}$$

Terms *a*, *b*, *c*, and *d* are defined by a suite of parameters from previously described biomolecule definitions. For an extensive description of terms *a*, *b*, and *c*, derivations, and parameter definitions, please refer to the supplementary material (Table S3 and S4). Here, the major difference between the two equations is that solving for phosphorus requires a cubic, rather than a quadratic, function. This occurs due to the additional growth rate factor (Eq. 4) necessary to quantify the uptake rate of phosphorus into the cell.

Similar to the Monod optimization, we used the Metropolis-Hastings algorithm with CFM-Phyto to converge to the best representation of the data, predicting the best values for the affinity to phosphorus (*A_P_*) and the stoichiometric ratio for the cellular photosynthetic enzyme nitrogen to chlorophyll ratio (*A_Pho_*; Table S5). We also included the values for *A*_*P*_ (or *A*_*N*_, affinity to nitrogen (27), for a specific case) and *A*_*Pho*_ for the example cases in Table S5. Phosphorus concentration does not directly influence this parameter, *A_Pho_*, rather it indirectly affects the enzyme due to the changes in RNA concentration, which comprises a large fraction of intracellular phosphorus. Additionally, we defined a set light intensity (I) for the model runs which was equal to the respective light intensity used in each experiment.

## Data Availability

The model code for this study can be found here: https://zenodo.org/badge/latestdoi/606055092

## SUPPLEMENTAL MATERIAL

Supplemental material msystems.00611-23-s0001.docx Supplemental figures and tables.

ASM does not own the copyrights to Supplemental Material that may be linked to, or accessed through, an article. The authors have granted ASM a non-exclusive, world-wide license to publish the Supplemental Material files. Please contact the corresponding author directly for reuse.
