# Supplementary material for: Saturating growth rate against phosphorus concentration explained by macromolecular allocation
Source: mSystems. 2023 Aug 29;8(5):e00611-23. doi: 10.1128/msystems.00611-23 (PMC10654069; doi:10.1128/msystems.00611-23)
Supplement: Supplemental material — Supplemental figures and tables. [file msystems.00611-23-s0001.docx]

| 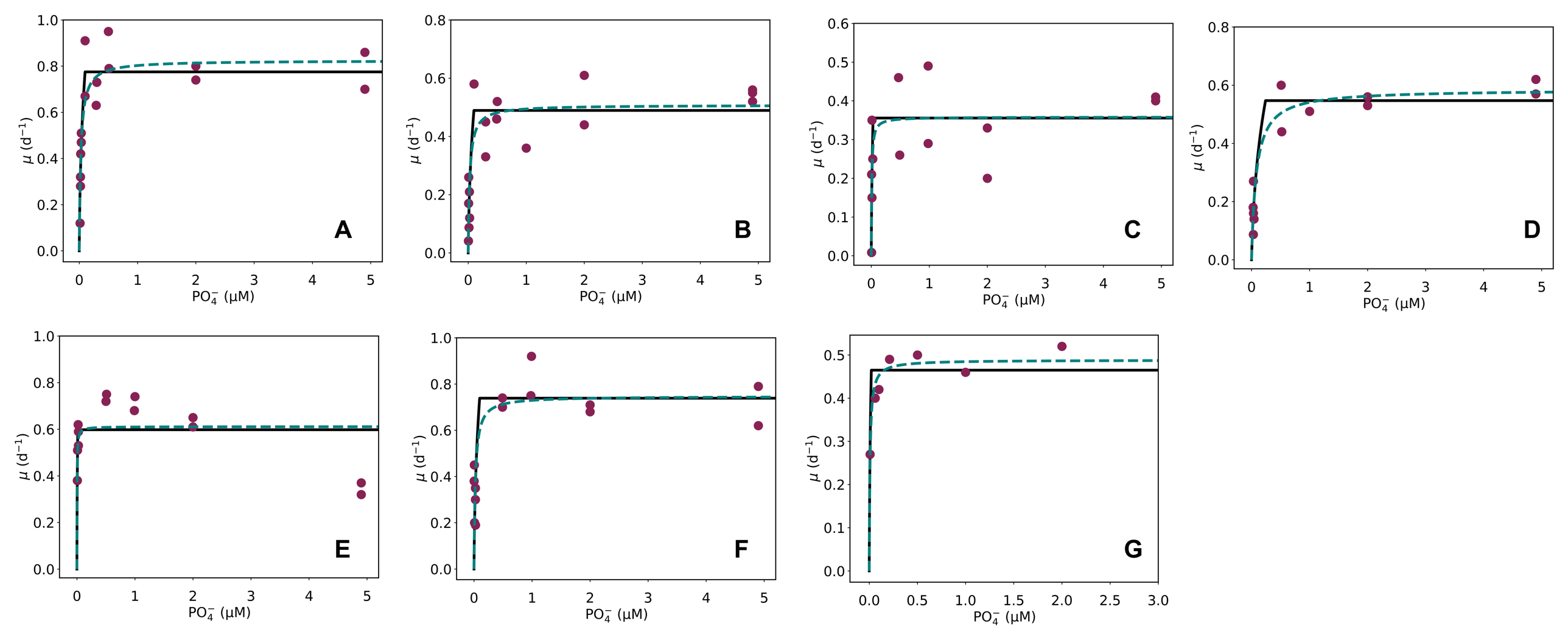 |
| --- |
| Figure S1. Modeled representations of growth rate vs PO_4_^3-^ concentration. Dots are data, dotted teal curves are Monod predictions, and continuous black lines are Cell Flux Model predictions. (A) *Chlamydomonas sp.* (1) (B) *Cryptomonas sp.* (1) (C) *Nitzschia acicularis* (1) (D) *Nitzschia linearis* (1) (E) *Synedra radians* (1) (F) *Synedra rumpens* (1) (G) *Synechococcus sp.* (2) |

| 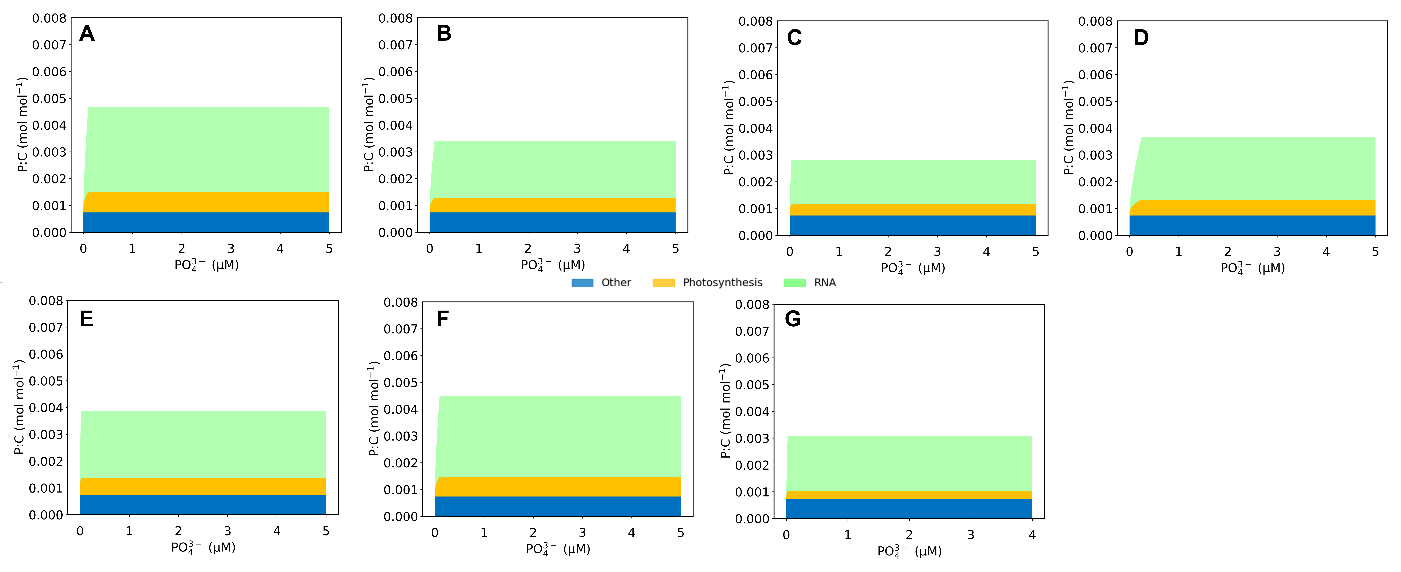 |
| --- |
| Figure S2. Species-specific predictions of macromolecular allocation of P to 3 cellular pools : RNA (green), photosynthetic molecules (orange) and other (blue). Other molecules include DNA and remaining P. (A) *Chlamydomonas sp.* (1) (B) *Cryptomonas sp.* (1) (C) *Nitzschia acicularis* (1) (D) *Nitzschia linearis* (1) (E) *Synedra radians* (1) (F) *Synedra rumpens* (1) (G) *Synechococcus sp.* (2) |

| Table S1. Sources of datasets with key details from the experimental set-up used to inform our model and the Monod mathematical model. |
| --- |
| \| Source \| Phytoplankton \| Light intensity reported \| Nutrient information \| Note \| \| --- \| --- \| --- \| --- \| --- \| \| (3)  Ghaffar et al., 2017 \| *Microcystis* \| 30 μmol m^-2^ sec^-1^ \| 0.5, 1, 2, 4, 8, 16, 32, 64, 128  and 256 μg PO_4_^-^P L^-1^. \| Ten treatments of varying nutrient levels \| \| (1)  Grover, 1989 \| *Chorella sp*. *Nitzschia palea* *Oocystis pusilla* *Scenedesmus quadricauda* *Sphaerocystis schroeteri*  *Chlamydomonas sp.*  *Cryptomonas sp.*  *Nitzschia acicularis* *Nitzschia linearis*  *Synedra radians* *Synedra rumpens* \| 60 μmol m^-2^ sec^-1^ \| Initial P concentration=0.24 μmol L^-1^ \|  \| \| (4)  Kim et al., 2015 \| *Synechocystis sp.* PCC6803 \| 82.8 μmol m^-2^ sec^-1^ \|  \|  \| \| (2)  Timmermans et al., 2005 \| *Pelagomonas capsulatus*  *Synechococcus sp.* \| 120 μmol m^-2^ sec^-1^ \| 0.0 (blank), +0.05,  +0.1, +0.2, +0.5, +1.0x10^-6^ M PO_4_^3-^ \|  \| |

| Table S2. Resultant maximum growth rates ($\mu_{max}$), half-saturation constants (K_S_), and prediction of fit to data from Monod kinetics optimization. |
| --- |
| \| Figure Number \| $\mu_{max}$ (d^-1^) \| K_S_ (μM) \| \| --- \| --- \| --- \| \| 1A \| 0.313 \| 5.418 \| \| 1B \| 0.813 \| 0.033 \| \| 1C \| 0.961 \| 0.094 \| \| 1D \| 0.551 \| 0.067 \| \| 1E \| 0.827 \| 0.067 \| \| 1F \| 0.517 \| 0.030 \| \| 1G \| 1.607 \| 0.078 \| \| 1H \| 1.679 \| 0.092 \| \| S1A \| 0.844 \| 0.027 \| \| S1B \| 0.430 \| 0.028 \| \| S1C \| 0.390 \| 0.008 \| \| S1D \| 0.864 \| 0.083 \| \| S1E \| 0.595 \| 0.094 \| \| S1F \| 0.811 \| 0.003 \| \| S1G \| 0.504 \| 0.008 \| |

| Table S3. Detailed description of *a*, *b*, *c* terms. |
| --- |
| \| Under C limitation:  $0=a_{C}\mu^{2}+b_{C}\mu+c_{C}$  where  $a_{C}=Y_{RNA}^{C:P}A_{RNA}^{P}(A_{pho}A_{Chl}\left( I \right)+A_{Bio})$  $b_{C}=\left( 1+A_{Pho}+Y_{Plip}^{C:P}A_{Pho}^{P:Chl} \right)A_{Chl}\left( I \right)+A_{Bio}+Y_{RNA}^{C:P}A_{RNA}^{P}(A_{pho}B_{Chl}\left( I \right)+Q_{C}^{Pro-Other})$  $c_{C}=\left( 1+A_{Pho}+Y_{Plip}^{C:P}A_{Pho}^{P:Chl} \right)B_{Chl}\left( I \right)+Q_{C}^{Other}+Y_{RNA}^{C:P}Q_{P,min}^{RNA}-1$ \| [eq S1] \| \| --- \| --- \| \| Under P limitation  0= $a_{P}\mu^{3}+b_{P}\mu^{2}+c_{P}\mu+d_{P}$  where  $a_{P}=A_{RNA}^{P}(A_{Pho}A_{Chl}\left( I \right)+A_{Bio})$  $b_{P}=A_{Pho}^{P:Chl}A_{Chl}\left( I \right)+A_{RNA}^{P}(A_{Pho}B_{Chl}\left( I \right)+Q_{C}^{Pro-Other})$  $c_{P}=Q_{P}^{Other0}+A_{Pro}^{P:Chl}B_{Chl}\left( I \right)+Q_{P,min}^{RNA}+Y_{DNA}^{P:C}Q_{C}^{DNA}$  $d_{P}=-V_{P}$=${-A}_{P}[{PO}_{4}^{3-}]$ \| [eq S2] \|   Here P storage is excluded from our equation to simulate phosphorus limitation here and it does not affect the growth rate even under C limitation. We do not include N storage as it does not affect the growth rate of the cell. Solution for C limitation is identical to that in (equation 29 in Inomura et al 2020). The solution under P limitation is obtained from [eq 4, 5] using the expression of *Q_P_* (P:C) without P storage in (equation 37 in Inomura et al 2020). Descriptions for *A_Chl_*(*I*) and *B_Chl_*(*I*) are in Inomura et al 2020. |

| Table S4. List of definitions and units for Table S4 |
| --- |
| \| Parameter \| Definition \| Units \| \| --- \| --- \| --- \| \| $Y_{Plip}^{C:P}$ \| Stoichiometric ratio carbon to phosphorus in phospholipids in thylakoid membrane \| mol C mol P^-1^ \| \| $A_{Pho}^{P:Chl}$ \| Constant of proportionality relating chlorophyll to the thylakoid membranes \| mol P mol C^-1^ \| \| $A_{Chl}\left( I \right)$ \| Growth rate dependent term for photosynthetic rate \| mol C mol C^-1^ d \| \| $B_{Chl}(I)$ \| Growth rate independent term for photosynthetic rate \| mol C mol C^-1^ \| \| $\mu$ \| Growth rate \| d^-1^ \| \| $Y_{RNA}^{C:P}$ \| Stoichiometric ratio carbon to phosphorus in RNA molecules \| mol C mol P^-1^ \| \| $A_{P}^{RNA}$ \| Constant of proportionality relating protein, growth rate and RNA molecules \| mol P mol C^-1^d \| \| $Q_{P,min}^{RNA}$ \| The minimum amount of phosphorus in RNA molecules \| mol P mol C^-1^ \| \| $Q_{C}^{Pro-Oth}$ \| Amount of carbon in other (non-synthetic) proteins \| mol C mol C^-1^ \| \| $A_{Bio}$ \| Constant of proportionality relating growth rate and biosynthetic protein \| mol C mol C^-1^ d \| \| $A_{Pho}$ \| Constant of proportionality relating chlorophyll and photosynthetic protein \| mol C (mol C in Chl)^-1^ \| \| $Q_{C}^{DNA}$ \| Amount of carbon in DNA molecules \| mol C mol C^-1^ \| \| $Q_{C}^{Csto}$ \| Amount of carbon in carbon storage \| mol C mol C^-1^ \| \| $Q_{C}^{Other}$ \| Amount of other carbon within the cell \| mol C mol C^-1^ \| \| $Q_{P}$ \| Amount of cellular phosphorus per C \| mol P mol C^-1^ \| \| $Q_{P}^{Other0}$ \| Amount of other phosphorus within the cell \| mol P mol C^-1^ \| \| $Y_{DNA}^{P:C}$ \| Stoichiometric ratio of phosphorus to carbon in DNA \| mol P mol C^-1^ \| \| $V_{P}$ \| Uptake rate of phosphate into the cell \| mol P mol C^-1^ d^-1^ \| \| $A_{P}$ \| Affinity constant for phosphate \| mol P mol C^-1^ d^-1^ µM^-1^ \| |

Except for *V_P_* and *A_P_*, notations are identical to those in Inomura et al 2020.

Table S5. Resultant (*A_Pho_*), (*A_P_*), and prediction of fit to data from CFM optimization.

| \| Figure Number \| *A_Pho_* (mol C (mol C in Chl)^-1^) \| *A_P_* (mol P mol C^-1^ d^-1^ µM^-1^) \| \| --- \| --- \| --- \| \| 3A \| 43.67253328 \| 4.01E-05 \| \| 3B \| 15.88689086 \| 3.07E-02 \| \| 3C \| 14.66951931 \| 7.34E-03 \| \| 3D \| 22.22525527 \| 3.95E-03 \| \| 3E \| 20.49136993 \| 1.25E-02 \| \| 3F \| 25.07639454 \| 1.34E-02 \| \| 3G \| 12.80218098 \| 1.86E-03 \| \| 3H \| 33.88449798 \| 6.29E-03 \| \| S2A \| 16.15530492 \| 3.61E-02 \| \| S2B \| 24.52799132 \| 1.69E-02 \| \| S2C \| 31.79828834 \| 3.18E-02 \| \| S2D \| 22.27338187 \| 8.29E-03 \| \| S2E \| 20.57599224 \| 1.51E-01 \| \| S2F \| 16.91573401 \| 3.41E-02 \| \| S2G \| 38.49799951 \| 6.62E-02 \| \| 3A,5A \| 15.98885249 \| 5.00E-03 \| \| 5B \| 15.98885249 \| A_N_ =5.00E-02^*^ \| \| 6A-Phosphorus \| 15.98885249 \| 5.00E-03 \| \| 6B-Phosphorus \| 15.98885249 \| 1.00E-02 \| \| 6A,6B-Nitrogen \| 15.98885249 \| A_N_ =5.00E-02^*^ \| |
| --- | --- | --- | --- | --- | --- | --- | --- | --- | --- | --- | --- | --- | --- | --- | --- | --- | --- | --- | --- | --- | --- | --- | --- | --- | --- | --- | --- | --- | --- | --- | --- | --- | --- | --- | --- | --- | --- | --- | --- | --- | --- | --- | --- | --- | --- | --- | --- | --- | --- | --- | --- | --- | --- | --- | --- | --- | --- | --- | --- | --- | --- | --- | --- |

^*^Values for nitrogen-related parameters (A_N_) come from previously published work (Kim et al., 2022). Units for A_N_ are mol N mol C^-1^ d^-1^ µM^-1^.

**Reference**

1. Grover JP. 1989. Phosphorus-dependent growth kinetics of 11 speciesof freshwater algae. Limnol Oceanogr 34:341-348.

2. Timmermans KR, van der Wagt B, Veldhuis MJW, Maatman A, de Baar HJW. 2005. Physiological responses of three species of marine pico-phytoplankton to ammonium, phosphate, iron and light limitation. Journal of Sea Research 53:109-120.

3. Ghaffar S, Stevenson RJ, Khan Z. 2017. Effect of phosphorus stress on *Microcystis aeruginosa* growth and phosphorus uptake. PLoS One 12:e0174349.

4. Kim H-W, Park S, Rittmann BE. 2015. Multi-component kinetics for the growth of the cyanobacterium *Synechocystis* sp. PCC6803. Environ Eng Res 20:347-355.

5. Inomura, K, Omata, AW, Talmy, D, Bragg, J, Deutsch, C, Follows, MJ. 2020. A Mechanistic Model of Macromolecular Allocation, Elemental Stoichiometry, and Growth rate in Phytoplankton. Frontiers in Microbiology 11:86.

6. Kim, J., Armin, G. & Inomura, K. Saturating relationship between phytoplankton growth rate and nutrient concentration explained by macromolecular allocation. *Curr. Res. Microb. Sci.* **3**, 100167 (2022).
